# Relationship between mindfulness, stress, and performance in medical students in pediatric emergency simulations

**DOI:** 10.3205/zma001474

**Published:** 2021-04-15

**Authors:** Kacper Łoś, Jacek Chmielewski, Grzegorz Cebula, Tomasz Bielecki, Kamil Torres, Włodzimierz Łuczyński

**Affiliations:** 1 Medical University of Białystok, Department of Medical Simulations, Białystok, Poland; 2 Medical University of Białystok, Department of Psychiatry, Białystok, Poland; 3 Jagiellonian University Medical College, Department of Medical Education, Kraków, Poland; 4 Medical University of Lublin, Department of Didactics and Medical Simulations, Lublin, Poland

**Keywords:** medical education, mindfulness, pediatric emergency medicine, medical simulation, non-technical skill

## Abstract

**Objectives: **Pediatric teams of emergency departments work under extreme stress, which affects high-level cognitive functions, specifically attention and memory. Therefore, the methods of stress management are being sought. Mindfulness as a process of intentionally paying attention to each moment with acceptance of each experience without judgment can potentially contribute to improving the performance of medical teams. Medical simulation is a technique that creates a situation to allow persons to experience a representation of a real event for the purpose of education. It has been shown that emergency medicine simulation may create a high physiological fidelity environment similarly to what is observed in a real emergency room. The aim of our study was to determine whether the technical and non-technical skills of medical students in the course of pediatric high fidelity simulations are related to their mindfulness and stress.

**Participants and methods:** A total of 166 standardized simulations were conducted among students of medicine in three simulation centers of medical universities, assessing: stress sensation (subjectively and heart rate/blood pressure), technical (checklists) and non-technical skills (Ottawa scale) and mindfulness (five facet mindfulness questionnaire): ClinicalTrials.gov ID: NCT03761355.

**Results: **The perception of stress among students was lower and more motivating if they were more mindful. Mindfulness of students correlated positively with avoiding fixation error. In the consecutive simulations the leaders’ non-technical skills improved, although no change was noted in their technical skills.

**Conclusion: **The results of our research indicate that mindfulness influence the non-technical skills and the perception of stress of medical students during pediatric emergency simulations. Further research is needed to show whether mindfulness training leads to improvement in this field.

## Zusammenfassung

**Ziele: **Pädiatrische Teams arbeiten in Notfallabteilungen unter extremem Stress, der hochkognitive Funktionen, insbesondere Aufmerksamkeit und Gedächtnis, beeinflusst. Daher wird nach Methoden des Stressmanagements gesucht. Achtsamkeit ist ein Prozess, bei dem jedem Moment bewusst Beachtung geschenkt wird, jede Erfahrung ohne Beurteilung angenommen wird, was potenziell zu einer Verbesserung der Leistung ärztlicher Teams beitragen kann. Medizinische Simulation ist eine Technik, in der eine Situation geschaffen wird, in der Personen die Möglichkeit gegeben wird, zu Ausbildungszwecken Erfahrungen in Form der Darstellung eines echten Ereignisses zu machen. Es hat sich gezeigt, dass in Notfallmedizinsimulationen eine sehr genaue physiologische Umgebung geschaffen werden kann, die ähnlich dem Geschehen ist, was in einer echten Notfallabteilung beobachtet wird. Das Ziel unserer Studie war es, festzustellen, ob die fachlichen bzw. nichtfachlichen Fähigkeiten von Studierenden der Medizin im Verlauf der pädiatrischen hochgenauen Simulationen eine Beziehung zu ihrer Achtsamkeit und ihrem Stress aufweisen.

**Teilnehmer und Methoden: **Es wurden insgesamt 166 standardisierte Simulationen mit Medizinstudenten in drei Simulationszentren an medizinischen Hochschulen durchgeführt, bei denen Stressempfinden (subjektiv und Herzfrequenz/Blutdruck),

fachliche (Checklisten) und nichtfachliche Fähigkeiten (Ottawa-Skala) sowie Achtsamkeit (Fünf-Facetten-Achtsamkeitsfragebogen), Clinical Trials.gov ID: NCT03761355, untersucht wurden.

**Ergebnisse: **Die Wahrnehmung von Stress seitens der Medizinstudenten war geringer und motivierender, wenn sie achtsamer waren. Die Achtsamkeit der Studierenden korrelierte positiv mit der Vermeidung von Fixation Errors. In den fortlaufenden Simulationen verbesserten sich die nichtfachlichen Fähigkeiten der Teilnehmer, jedoch wurde keine Veränderung der fachlichen Fähigkeiten festgestellt.

**Schlussfolgerung: **Die Ergebnisse unserer Forschung deuten darauf hin, dass Achtsamkeit nichtfachliche Fähigkeiten und die Wahrnehmung von Stress bei Medizinstudenten in pädiatrischen Notfallsimulationen beeinflusst. Weitere Forschungen sind nötig, um zu zeigen, ob Achtsamkeitstraining zu Verbesserungen in diesem Bereich führt.

## 1. Introduction

Pediatric emergency department staff work in intense, chaotic and unpredictable environments and have been shown to have the highest levels of psychosocial distress among all healthcare providers [1]. Stress affects high-level cognitive functions, specifically attention and memory, and this increases the already high stakes for young doctors. The effects of stress can have both enhancing and impairing effects on learning and memory [2]. Lapses in attention increase the risk of serious consequences such as medical errors, failure to recognize life-threatening signs and symptoms, and other essential patient safety issues [3]. Therefore, the methods of stress management are being sought. One of the candidates may be mindfulness. 

Mindfulness is a process of intentionally paying attention to each moment with curiosity, openness and acceptance of each experience without judgment [4]. This is achieved through an attitude of lack of valuation, clarity, acceptance, patience, sincerity, unexpectedness, loving kindness, care and compassion for the current situation. The goal of mindfulness is to empower individuals to respond to situations consciously rather than automatically. All these features can have a potentially positive effect on medical education. Being mindful is associated with decreased stress, anxiety, depression, improved mood, self-empathy and empathy among medical students [5]. So it is possible that mindfulness could enhance medical students’ capacity for focused attention and concentration by increasing present moment awareness in pediatric emergency cases. Certainly mindfulness is a major strategy to enhance wellness in emergency medicine residency training programs [6].

Medical simulation is a technique that creates a situation or environment to allow persons to experience a representation of a real event for the purpose of practice, learning, evaluation, testing or to gain understanding of systems or human actions [7]. It has been shown that emergency medicine simulation may create a high physiological fidelity environment similarly to what is observed in a real emergency room [8]. We can teach skills, clinical decision making, communication and teamwork in an environment that is safe for the patient and the student (“psychological reality and safety”) [9]. Simulation scenarios cover technical (e.g. diagnostic and therapeutic procedures) and non-technical skills. Non-technical competences are the skills of communication, leadership, teamwork, situational awareness, decision-making, resource management, safe practice, adverse event minimization, and professionalism, also known as behavioral or teamwork skills [7]. Life-threatening situations are less frequent in pediatric than in adult emergencies, and also requirements for non-technical and technical skills may be stronger in pediatrics and neonatology than in other fields of healthcare [10]. Therefore the high-fidelity simulation should be used to teach practically all aspects of pediatric acute care [11].

No research has been performed concerning the association between being mindful, stress and performance in emergency medicine under standardized conditions. The aim of our work was to test the hypothesis that technical and/or non-technical skills of medical students during pediatric emergency simulations are related to their mindfulness and stress. In general, we anticipated that the results of our study could lead to better understanding of the mechanisms that influence performance of medical students during pediatric emergency cases and may enable them to improve their skills in their future professional life.

## 2. Participants and methods

### 2.1. Participants

**Figure 1:**
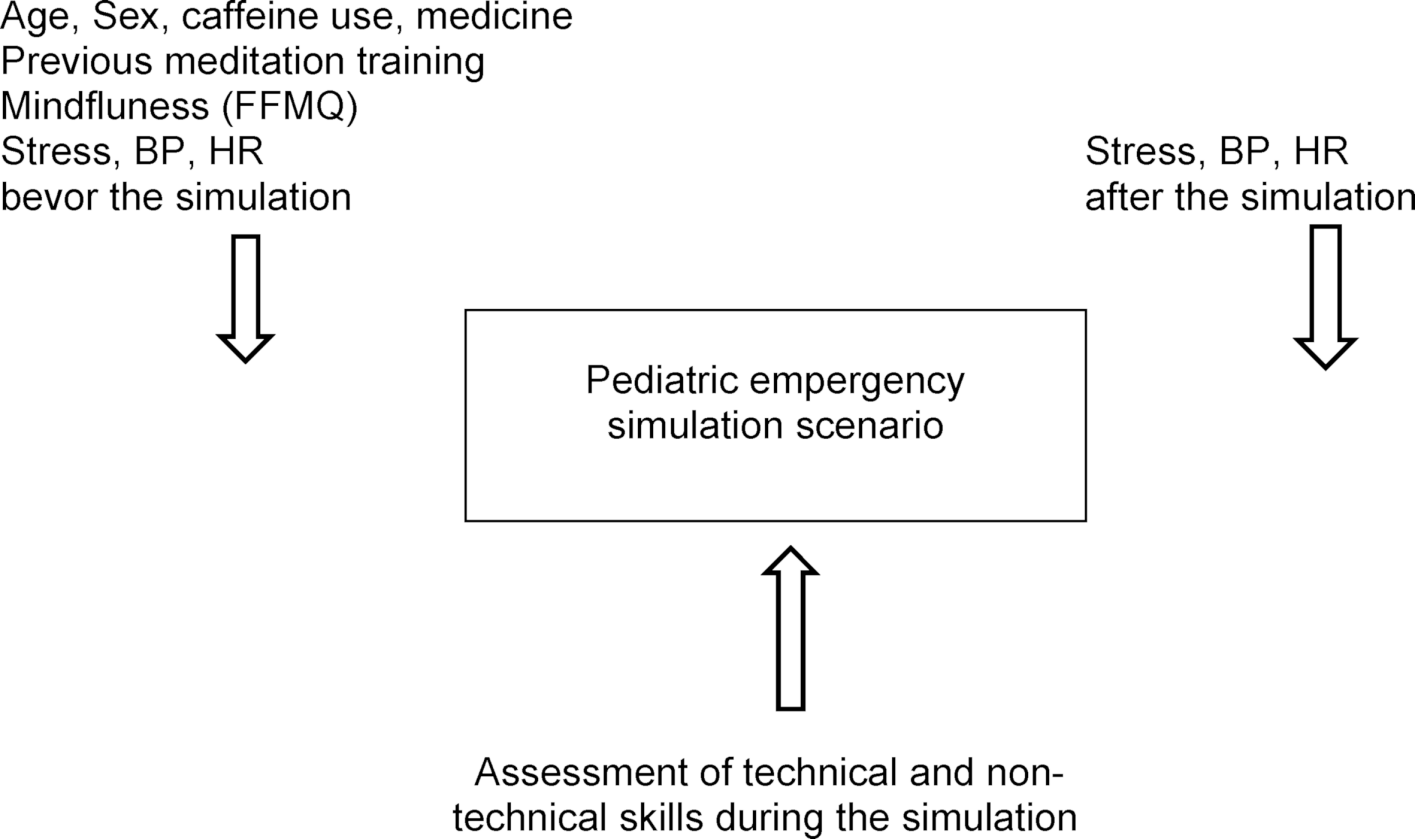
Design of the study – flow diagram of the methods used to assess medical students’ stress, mindfulness and performance before and after pediatric emergency simulations.

The project was planned as an observational cohort study in a group of graduating medical students (Clinical Trials.gov ID: NCT03761355). The research was conducted between October 2017 and October 2018 in three Polish medical simulation centers. The inclusion criterion was being a graduating student of medicine and consent to participate in the study. The exclusion criterion was pregnancy. The design of the study is presented on figure 1 (Fig. 1). 

#### Medical simulations

The simulations were constructed as high-fidelity scenarios in life-threatening situations in children (topics: supraventricular tachycardia, febrile convulsions, bronchial asthma, ketoacidosis, anaphylactic shock, paracetamol intoxication). They started in the morning and were identical for all groups of students (the same introduction to the simulator and medical equipment based on checklists and the same order of the scenarios). The level of task difficulty was intermediate and was evaluated based on the pilot simulations with both students and young doctors. Each scenario had two equal goals - technical and non-technical e.g treatment of exacerbation of asthma due to pnemonia and avoiding fixation error (i.e. not only asthma but also pnemonia as a cause of the poor general condition of the child). During the implementation of the simulation scenario, the students played different roles (team manager, member of the medical team or actor – the patient's caregiver). The analysis concerns only the scenarios in which the students acted as team leaders (skills and interactions with the other participants of the simulation were assessed).

The following data of medical students were assessed: age, sex and the fact of participating in mindfulness training or other secular or religious meditations. Stress and its impact on simulation were assessed both subjectively by participants (in a five-step scale from mobilizing to discouraging; the higher the score, the greater or more discouraging the stress, maximum 5 points) as well as by assessing the heart rate and blood pressure. Medicines taken and the amount of caffeine consumed by students every day and before the simulation were also noted, as well as the subsequent number of the simulation on a given day and during the entire medical studies (our students start simulation cirriculum at 4^th^ year of the faculty).

#### 2.2. Technical and non-technical skills

Technical skills were assessed on the basis of checklists designed for each scenario. The assessment was divided into an interview, physical examination, diagnosis and treatment. The more points on the scale, the better the technical skills (maximum 10 points).

Non-technical skills were assessed by the Ottawa Crisis Resource Management Global Rating Scale (*Ottawa GRS*) and checklist [12], [13]. The Ottawa GRS, an instrument developed for crisis resource management skill assessment, was designed for acute care physicians and is also widely used in medical simulation. It has well-defined rating scales for each of its categories: leadership skills (stays calm and in control during crisis, prompt and firm decision-making), situational awareness (avoids fixation error), communication skills (communicates clearly and concisely, uses directed verbal /non-verbal communication, listens to team input), problem solving (organized and efficient problem solving approach, considers alternatives during crisis), resource utilization (calls for help appropriately, utilizes resources at hand) [12]. Each category is measured on a seven-point anchored ordinal scale with descriptive anchors to provide guidelines on alternating points along the scale. These descriptions are added to reduce personal bias in interpreting performance. The higher the Ottawa GRS score, the better the non-technical skills (maximum 42 points).

Prior to the research protocol all instructors were trained in the assessment of non-technical skills by GC and KT – international experts in this field. Non-technical skills were assessed by two independent instructors/observers during each simulation in each center. All the instructors had more than two years of work experience in the simulation center, performing and assessing at least 50 simulations of high-fidelity each year. They were all trained for the minimum of 3 years in simulation and debriefing. The inter-rater coefficient of variation was 5% for the assessment of non-technical skills. Mean scores between the two observers were used as the reference value.

#### 2.3. Mindfulness

Mindfulness was assessed before the simulations according to the short version of Five Facet Mindfulness Questionnaire (FFMQ) in Polish adaptation and validation [14], [15]. FFMQ is used to measure the depth of mindfulness, that is, the specific state of attention, which is the result of constantly directing attention, in the non-evaluative and non-judgmental way, to what is happening at the present moment. FFMQ evaluates five factors: conscious presence, non-reactivity, non-judgment, observation and description. The higher the FFMQ score, the higher the level of mindfulness.

The study design was approved by the Ethics Committee at the Medical University of Bialystok in accordance with the Declaration of Helsinki (No R-I-002/358/2017). Signed informed consent was obtained from students. The rates of consent was 94.9%. The main reason for the consent refusal was lack of time to complete the survey. Students who agreed to participate in the study and those who did not give their consent did not differ in sex, age or in technical and non-technical skills assessment scores.

Data are presented as means and standard deviation (SD) and rates of incidence of a given characteristic in the evaluated group of students. Univariate analysis was conducted using the Mann-Whitney U test for continuous variables and the Chi-square test for the nominal ones. Spearman's rank correlation coefficient was used to evaluate the relationships between mindfulness, stress and performance of medical students in simulations. A p<0.05 was considered statistically significant. Statistical analysis was performed using the Statistica 13 software (StatSoft, Tulsa, OK, USA). Only students with all data available were included in the analysis.

## 3. Results

In total, 166 students were qualified for the study. As each student played a role of a team leader in a pediatric emergency situation at least once, 166 simulations were analyzed. Data referring to age, sex, meditation/prayer, use of caffeine and medicines, and stress before and after simulations are shown in table 1 (Tab. 1). The average results of students on the scales of technical and non-technical skills and mindfulness are presented in table 2 (Tab. 2). Relationships between mindfulness, performance and stress of medical students in pediatric emergency simulations – Spearman's rank correlation coefficient – are shown in table 3 (Tab. 3).

### 3.1. Stress and students’ mindful presence

The students' perception of stress before and during simulation correlated with their mindfulness. The lower perception level of stress before simulation was related to total mindfulness, lower reactivity and lower level of judgment (see table 3 (Tab. 3)). In addition, stress appearing during simulation was perceived as more mobilizing if the students were characterized by more conscious presence and higher level of description of the present moment (see table 3 (Tab. 3)).

Participation in meditations/prayers did not affect the other parameters assessed in the study. No correlation was also observed in the use of caffeine permanently and before simulation in relation to pre-simulation stress, the heart rate and blood pressure before and after simulation. No differences were found between meditating and non-meditating students in the mindfulness scale (non-reactivity, observation, conscious action, describing and not judging).

#### 3.2. Stress and performance of medical students

The stress experienced before a simulation influenced some of the parameters on the non-technical skills scale (the Ottawa GRS). There was a positive correlation between the subjective feeling of stress before the simulation and the obtained results in terms of team management and communication in the team (see table 3 (Tab. 3)); the perceived stress was not related to technical skills assessed by a dedicated, standardized checklist.

The total outcome of students in technical skills was not correlated with the total score obtained in non-technical skills. However, a situational awareness (the Ottawa GRS) was associated with a higher score in technical skills (see table 3 (Tab. 3)).

The analysis of all completed simulations showed that all non-technical skills improved along with the number of high-fidelity medical simulations performed by a given team (r=0.33, p<0.001). This correlation was not observed in the case of technical skills (p>0.05).

A comparison of the Ottawa GRS scores showed statistically significantly lower average scores for situational awareness skills (fixation error) than for other non-technical skills of medical students (p <0.001) (see table 2 (Tab. 2)). Situational awareness skills did not correlate with stress, using caffeine, medications or participating in meditations/prayers.

#### 3.3. Mindfulness and performance

The results of the students obtained in checklists in the area of technical skills did not correlate with their mindfulness (p>0.05). Similarly, the FFMQ scale total score was not related to the total score obtained using the Ottawa GRS for non-technical skills. However, avoiding fixation error (situation awareness skills) was positively associated with conscious action on the mindfulness scale (see table 3 (Tab. 3)). Mindfulness of students did not correlate with a change in the level of perceived stress, or alterations in heart rate and pressure before and after a simulation.

## 4. Discussion

In standardized conditions of pediatric high-fidelity simulations, we searched for links of conscious presence with technical and non-technical skills as well as the perception of stress by medical students. The stress was milder and more motivating if the students were more mindful. Mindfulness of students correlated positively with avoiding fixation error. The results of our research indicate the importance of mindfulness and stress in teamwork during simulated pediatric emergency cases. So far, no such research has been carried out, and the effect of mindfulness on physicians’ performance in emergency medicine, whether beneficial or not, was only hypothetical.

In our group of medical students the total score on the technical skills scale did not correlate with the overall result of the non-technical competencies. However, one of the soft skills components, i.e. greater situation awareness, resulted in a higher score on the technical skills scale. In similar conditions of pediatric simulations no direct relationship was found between situation awareness and goal achievement [16]. In that study, consensus on the primary problem (shared mental model) led to faster achievement of predefined goals. However, in other acute care simulations, non-technical skills significantly predicted students’ clinical performance as assessed by nurses [17]. In one of the studies, like in our group, repeating the simulation improved non-technical skills, such as communication, teamwork, situational awareness and decision making [18]. However, Clarke et al. showed that the only variable affecting the overall assessment of non-technical competencies during simulations (also evaluated by the Ottawa GRS) was the residents' progress in the specialization program (so called PGY status) or clinical experience rather than stress or its subjective feeling [19]. 

It is extremely difficult to perform research that can show the impact of mindfulness on patient care. In the present study we showed for the first time that mindfulness is associated with non-technical skill i.e. avoiding fixation error in pediatric emergency simulations. In our opinion, situation awareness (fixation error) was the weakest non-technical skill among all the skills assessed using the Ottawa GRS scale. During the pre-briefing, students often uttered a probable diagnosis aloud and were fixating on it during the simulation, without verifying the hypothesis, despite all data received (interview, physical examination, additional test results). Loss of situation awareness can lead to errors. On the other hand the Simulation-Based Crisis Resource Management training implemented among pediatric cardiac intensive care unit providers has improved situational awareness including reporting of doubts concerning the appropriate procedure to the team leader [20].

The FFMQ scores obtained by our medical students were similar to the results reported from the group of 800 Polish adults among whom the scale was validated, thus indicating that medical students are as mindful as other adult Poles [14]. 

In our observation, a higher level of mindfulness not only reduced the subjective perception of stress before simulation, but also correlated with its more motivating impact during the simulation scenario. The results of some studies indicate a connection between mindfulness and well-being or education and work in medical professions and in students of medical courses. Such mindfulness effects as reduced stress, increased self-regulation (the ability to effectively manage one's thoughts and actions to complete a task) can be particularly useful for medical students during pediatric emergency cases. A meta-analysis of 19 studies concerning the use of mindfulness in medical students showed that mindfulness-based interventions decrease stress, anxiety, depression and improve mindfulness, mood, self-empathy and empathy [5]. As revealed by the authors of that analysis, mindfulness training can be relatively easily adapted and integrated with modern teaching of medicine. A focus on the present moment can help young doctors concentrate on what is really important, which may refer both to helping patients and themselves. Mindfulness objective is to allow healthcare workers to keep a distance from a mentally and emotionally stressful environment. Mindfulness can also be helpful in teaching professionalism, including accepting one's own boundaries, priorities and being resilient. In one study, more mindful residents asked for help more often and seemed to be more open toward feedback [21]. In our simulations, one of the technical skills, in some scenarios, was to call a specialist for consultation. However, we found no connection between greater mindfulness and more frequent requests for help in this area. Learning critical thinking, developing cognitive and affective biases, using such processes as reflection and mindfulness can lead to greater creativity, lateral thinking, and innovation in the diagnosis and treatment of patients [22]. Perhaps the routine diagnostic and therapeutic process that does not always produce a desired effect should be enriched with the awareness of the inhibitors and facilitators of rationality in decision making. It seems that the simulation room is a very good place to learn mindfulness in diagnostic and therapeutic procedures before applying this technique in real medical life [23].

The strength of our study is the unique pediatric character of the simulation, a large number of participants and simulations, great experience of simulation centers involved in the project, lack of voluntary nature of the research and standardized conditions for study implementation. The limitations of our study are certainly the subjectivity and difficulty of the Ottawa GRS application for the non-technical skills. However, this tool is considered to be easier to use, more practical and more reliable than a similar instrument i.e. Anesthetists’ Non-Technical Skills (ANTS) behavioral marker system [13]. Our project also lacks self-assessment of students’ activities, and it would be extremely interesting to compare it with the assessment of instructors. However, our attempts to carry out such assessments failed: students were not able to evaluate their actions using quite complicated scales. Moreover, the outcome of our research conducted with medical students cannot fully translate into the real work of physicians, since the final effect involves a great deal of additional factors as well as professional environment or family, etc., which determine the attainment of mindfulness objectives in everyday work with patients in hospital or outpatient clinic. 

Does greater mindfulness indeed influence the actions of doctors in real medical situations? Should mindfulness be included in the university curriculum for medical students? Should mindfulness be taught as an elective or obligatory subject? It would be interesting to check when the interventions are optimal – at the beginning or end of the study, at the start or finish of the residency period? These and many other questions should be answered in future randomized trials.

## 5. Conclusions

The results of our research indicate a relationship between mindfulness of medical students and their non-technical skills and the perception of stress in simulations of pediatric emergency situations. Further research is needed to show whether mindfulness training leads to changes in this field. Perhaps mindfulness courses should be included in the medical university curriculum and early career of a young doctor to reduce stress and improve the diagnostic and therapeutic effectiveness of physicians working individually and in teams.

## Abbreviations

- ANTS – Anesthetists' Non-Technical Skills
- FFQM – Five Facet Mindfulness Questionnaire
- HRV – heart rate variability
- Ottawa GRS – Ottawa Crisis Resource Management Global Rating Scale

## Authors’ contributions

JC, WL, GC, TB and KT designed the study; JC and WL were a major contributors in writing the manuscript; WL, KL, TB and KT collected and analysed the patient’s data; all authors read and approved the final manuscript.

## Data availability

The raw data sets including data base (in Microsoft Access) used to support the findings of this study are available from the corresponding author upon request.

## Funding

The study was funded by Medical University of Białystok, Poland.

## Competing interests

The authors declare that they have no competing interests. 

## Figures and Tables

**Table 1:**
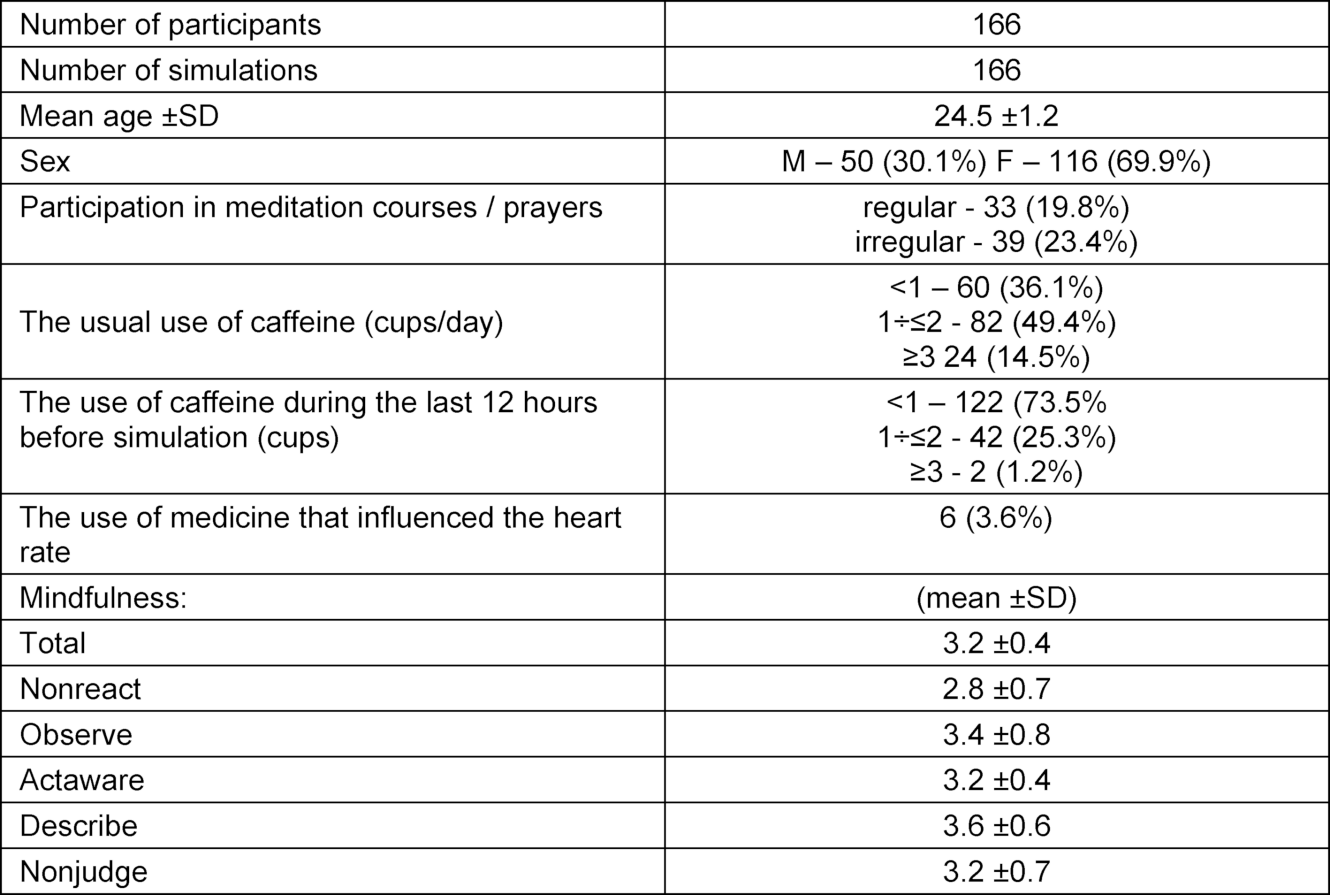
Data of medical students participating in pediatric simulations.

**Table 2:**
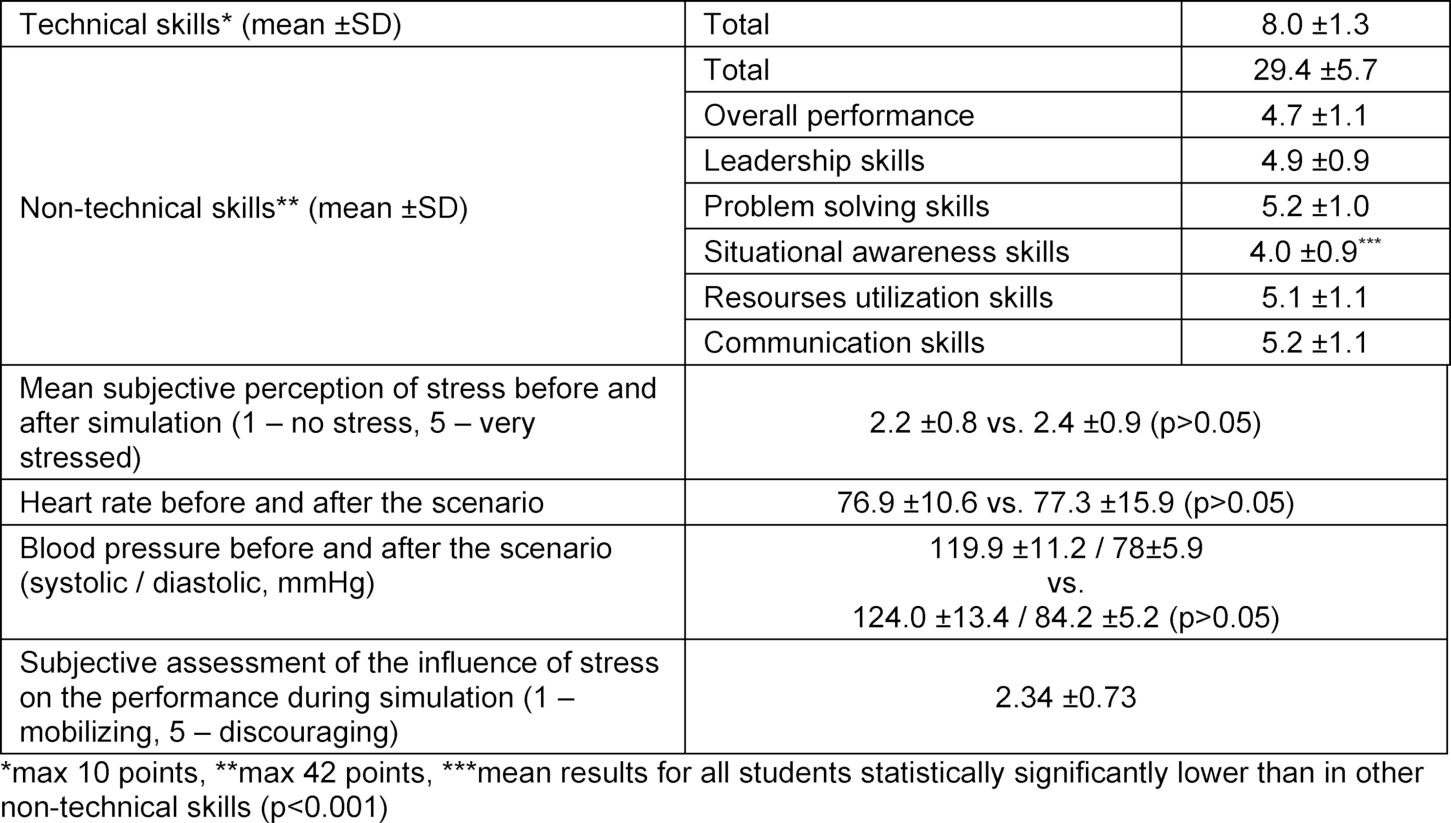
Results concerning technical, non-technical skills and mindfulness in medical students during pediatric emergency simulations.

**Table 3:**
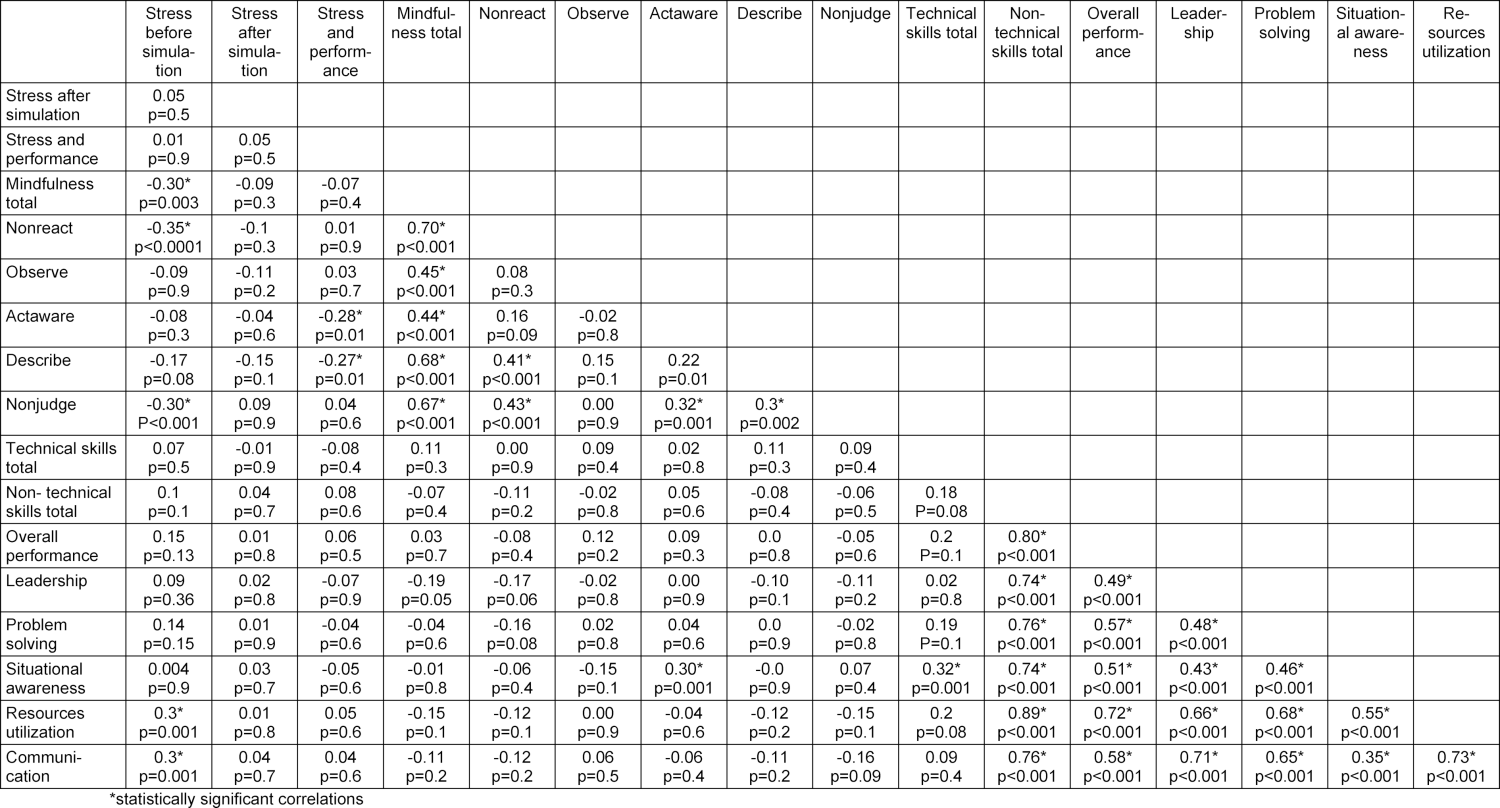
Relationships between mindfulness, performance and stress of medical students in pediatric emergency simulations.
